# Experiences and coping strategies among patients with Chronic Renal Failure (CRF) in Ghana: A phenomenological study

**DOI:** 10.1371/journal.pmen.0000279

**Published:** 2025-09-19

**Authors:** Emmanuel Abu Bonsra, Princess Yesutor Atsrim, Alex Korankye, Joyce Komesuor

**Affiliations:** 1 Department of Population and Behavioural Sciences, Fred N. Binka School of Public Health, University of Health and Allied Sciences, Hohoe, Ghana; 2 Family and Community Health, Fred N. Binka School of Public Health, University of Health and Allied Sciences, Hohoe, Ghana; 3 Department of Epidemiology and Biostatics, Fred N. Binka School of Public Health, University of Health and Allied Sciences, Hohoe, Ghana; Flinders University, AUSTRALIA

## Abstract

Globally, chronic renal failure (CRF) presents significant physical, emotional, and psychosocial challenges for patients. The burden of CRF is further compounded by limited access to specialized healthcare, financial constraints, and factors that shape patient experiences and coping strategies. Therefore, this study sought to explore the lived experiences and coping strategies of patients with chronic renal failure in Ghana. A phenomenological qualitative design was employed, using purposive. Data collection involved in-depth interviews, and the transcripts were analysed using thematic analysis with the aid of ATLAS.ti. The analysis was presented in a thematic network to visualize key themes and subthemes. The study did not adopt a pre-existing theoretical framework but was guided by participants’ narratives. Thematic analysis revealed two major themes: (1) Lived experiences of chronic renal failure, with subthemes of emotional distress, physical challenges, and financial hardship; and (2) Coping strategies, including emotional coping, avoidance, religious faith, and acceptance coping. The lived experiences of patients with chronic renal failure revealed profound emotional, physical, and financial challenges. Patients initially described their diagnosis as shocking and distressing, with many experiencing depression, anxiety, or suicidal thoughts. Financial strain and limited access to dialysis worsened their burden. Nonetheless, various coping strategies helped patients adapt and regain a sense of control. Acceptance, emotional and avoidance coping emerged as a powerful coping mechanism, where patients acknowledged their illness, adapted their lifestyle, and found resilience in living one day at a time. Living with chronic renal failure significantly impacts patients emotional, physical, and financial well-being, with many experiencing depression, anxiety, and suicidal thoughts. Despite these challenges, patients employ coping strategies such as emotional expression, avoidance, reliance on religious faith, and acceptance. The study emphasizes the need for the Ghana Health Service to prioritize individualized interventions, and post-diagnosis counselling, and to enhance coping effectiveness and improve patient outcomes.

## Introduction

### Background

Chronic Renal Failure (CRF), also known as chronic kidney disease (CKD), is a progressive and irreversible condition affecting millions globally [1]. An estimated 700 million people are currently affected by CKD, representing approximately 10% of the global population [2]. It is the third fastest-growing cause of death worldwide, with mortality increasing by 50% between 2000 and 2019 [3]. Projections indicate that by 2040, CKD will account for 5% of years of life lost (YLL), making it the fifth leading cause globally [4]. Despite its growing burden, only about 2 million people currently receive treatment through dialysis or transplantation, covering just 10% of those in need [5]. The global demand for kidney replacement therapy is expected to more than double from 2.6 million in 2010 to 5.4 million by 2030 [6].

Patients with CRF often endure significant physical, emotional, and psychosocial challenges that greatly affect their quality of life [7]. A systematic review of qualitative studies reports that individuals undergoing maintenance dialysis frequently experience disrupted life trajectories, psychological distress, and social isolation [8]. Common experiences include shock at diagnosis, severe physical symptoms, impaired occupational and social functioning, and shifts in identity and self-perception [8]. Emotional distress is heightened by disease uncertainty and treatment complexities, prompting patients to adopt various coping strategies [9]. These often begin with avoidant behaviour such as hiding their illness or surgical scars due to fear of stigma [10]. Patients also attempt to normalize their lives by distancing themselves mentally from the illness [9].

In low- and middle-income countries (LMICs), about 90% of people with CKD remain unaware of their condition, contributing to late diagnosis and placing additional strain on under-resourced health systems [2]. In Africa, rising rates of diabetes and hypertension the two leading causes of CKD are exacerbating this public health challenge [11]. Limited awareness, late detection, and high out-of-pocket treatment costs further complicate access to care, including dialysis services [11]. Consequently, CRF patients often suffer from depression and anxiety, which reduce overall well-being and quality of life [8].

However, patients on dialysis employ a variety of coping strategies to manage their condition [12]. These include seeking information, adhering to medication and dietary instructions, participating in purposeful activities, and engaging in clinical decisions to maintain a sense of control [13,14]. Others test boundaries by occasionally breaching dietary restrictions as a form of asserting autonomy [15]. While such coping mechanisms can promote resilience, they are not always sustainable. Many patients face alternating periods of emotional strength and vulnerability [12]. When these mechanisms fail, feelings of hopelessness may lead to emotional breakdown or, in extreme cases, suicidal ideation [8].

In Ghana, the estimated prevalence of CKD stands at 13.3%, higher than the global average [16]. Managing CRF is especially difficult due to the dual burden of treatment-related stress and systemic limitations [17]. Dialysis, while life-sustaining, demands long-term commitment and is financially burdensome [18]. Many patients face difficulties accessing regular treatment due to geographic barriers and out-of-pocket payment requirements [19]. This financial pressure, coupled with debilitating symptoms, significantly lowers patients’ quality of life [20].

Despite extensive clinical research on CKD in Ghana, there is limited understanding of the lived experiences of CRF patients. Specifically, the psychological burden, social isolation, financial strain, and coping mechanisms remain underexplored. This qualitative study seeks to fill this knowledge gap by exploring the lived experiences, coping strategies, and socio-emotional impacts among CRF patients receiving dialysis at selected teaching hospitals in Ghana. By foregrounding patients’ voices, this research aims to inform healthcare providers, policymakers, and support systems on tailored interventions. Therefore, this study sought to explore the lived experiences and coping strategies among patients with chronic renal failure in Ghana.

### Theoretical framework

This study is grounded in two complementary theories that provide a deeper understanding of how patients with chronic renal failure (CRF) experience and respond to stress: Lazarus and Folkman’s Transactional Model of Stress and Coping and the Interactive Stress Theory as employed by Abu Bonsra et al. [21,22].

Lazarus and Folkman’s model views stress as a dynamic process involving ongoing interaction between the individual and their environment [21]. Coping is shaped through two levels of cognitive appraisal: primary appraisal, where an individual assesses whether a situation poses a threat, and secondary appraisal, where they evaluate their resources and options to cope with the situation [21]. Coping strategies are then employed, typically as either problem-focused coping (addressing the stressor) or emotion-focused coping (managing emotional responses). This framework is particularly applicable to CRF patients who constantly evaluate their condition and make complex decisions about their care, lifestyle, and emotional well-being.

Building on this, the Interactive Stress Theory utilized by Abu Bonsra et al. [22] in their study on cancer patients in Ghana emphasizes the reciprocal relationship between the individual and contextual factors such as culture, spirituality, healthcare systems, and social networks. Unlike traditional stress theories that may isolate the individual’s cognitive processes, the Interactive Stress Theory considers how external systems and sociocultural realities shape coping responses over time. Abu Bonsra et al. found that patients’ coping strategies were not only internally motivated but also strongly influenced by cultural beliefs, religious values, family expectations, and institutional support systems [22]. In the present study, both frameworks are employed to capture the complex interplay between internal appraisals and external influences that shape the lived experiences of CRF patients. Lazarus and Folkman’s model informs the individual-level cognitive and emotional responses to diagnosis and treatment, while the Interactive Stress Theory provides a broader lens to explore how Ghanaian cultural, religious, and social contexts mediate these responses. This dual-theoretical foundation allows for a holistic interpretation of coping mechanisms, including both adaptive strategies (e.g., acceptance, faith-based coping) and maladaptive ones (e.g., avoidance, substance use), while also accounting for the structural and relational dynamics influencing patients’ experiences.

## Methods and materials

### Study setting

This study was conducted in three selected teaching hospitals in Ghana: Cape Coast Teaching Hospital (CCTH), Ho Teaching Hospital (HTH), and Komfo Anokye Teaching Hospital (KATH). Cape Coast Teaching Hospital (CCTH) is a key healthcare institution located in Cape Coast, Ghana. Affiliated with the University of Cape Coast, it plays a crucial role in the education and development of healthcare professionals. The hospital offers a broad range of medical services, including specialized care in Obstetrics & Gynaecology, Paediatrics, and Surgery. With a bed capacity of 400, CCTH serves as a regional referral center. In addition to its clinical services, it is a vital training ground for nursing and allied health students and is accredited to train resident doctors in Internal Medicine and Surgery.

Ho Teaching Hospital (HTH) is one of the five public teaching hospitals in Ghana. Initially constructed by Kaevener Construction International of the United Kingdom, the hospital was completed and handed over to the Government of Ghana in November 1998. It commenced operations on a small scale in April 1999 and was officially commissioned as the Volta Regional Hospital in December 2000 by former President John Jerry Rawlings and his wife. Strategically located, HTH provides specialized healthcare services to the Volta Region and neighbouring countries such as Togo, Benin, and Nigeria.

The hospital envisions itself as a Medical Tourist Centre, focusing on tertiary healthcare, medical education, and research. Komfo Anokye Teaching Hospital (KATH) is a leading healthcare institution situated in Kumasi, Ghana. As the second-largest hospital in the country, it serves as a tertiary referral center for the Ashanti Region and beyond. KATH is committed to providing high-quality healthcare and has become a focal point for stroke management, admitting over 1,000 stroke patients annually a significant increase from approximately 200 cases recorded 40 years ago. The hospital is equipped with modern medical facilities and specialized departments that cater to a wide range of health needs. Notably, KATH has a Stroke Thrombolysis Service, which administers clot-busting medications to patients experiencing acute ischemic strokes. This selection of hospitals provides a comprehensive representation of Ghana’s healthcare system, covering diverse geographical regions and medical specializations.

### Study design

This study employed a phenomenological qualitative research study design to explore the personal experiences and coping mechanisms of individuals with chronic kidney failure at Cape Coast Teaching Hospital (CCTH), Ho Teaching Hospital (HTH), and Komfo Anokye Teaching Hospital (KATH).

### Study population

The study population for this study consisted of adult CKD patients at the renal unit in the selected teaching hospitals, in Ghana.

### Inclusion criteria

Patients who were Adults (male and female) diagnosed with stage 5 chronic kidney disease (ESRD) who are currently undergoing dialysis and able to provide informed consent were included in the study.

### Exclusion criteria

Patients with end-stage kidney failure who are not on dialysis, as well as those who have undergone a transplant, were excluded from this study. Also, patients who were critically ill or unstable at the time of data collection.

### Sampling procedure

The hospitals were purposively selected for their status as major teaching and referral centers in different regions of Ghana, ensuring a diverse patient population. Cape Coast Teaching Hospital, Ho Teaching Hospital, and Komfo Anokye Teaching Hospital were chosen due to their specialized nephrology units, their role in providing tertiary healthcare, and their capacity to manage chronic kidney disease patients. A purposive sampling method was then employed to select adult patients with chronic kidney disease (CKD). Recruitment was carried out in collaboration with the nephrology departments of the hospitals to ensure a diverse representation of CKD stages and types. Initially, 140 participants were invited to participate in the study; however, 20 declined due to illness, communication barriers, or time constraints. The final sample consisted of 120 participants, at which point data saturation was achieved.

### Research team

The data collection process involved four researchers: the first (male), second (female), third (male) authors, and the fourth (male). All members had prior experience conducting qualitative interviews. To ensure consistency in the use of research tools and interviewing methods, a two-day training session was conducted for the research team.

### Data collection and materials

In-depth, face-to-face interviews were conducted with patients at the three teaching hospitals. The data collection process utilized an in-depth interview guide. An in-depth interview guide was used to maintain consistency across interviews while allowing participants the flexibility to express their experiences freely and explore emerging issues in depth. These tools were designed to gather detailed information on the emotional experiences of the participants. The interviews also explored the various coping strategies employed by individuals in managing their condition. Participants were encouraged to share personal insights into the challenges they faced during their health journey. The aim was to understand how the condition impacts their emotional well-being and how they navigate these difficulties. Additionally, the study sought to explore any patterns or commonalities in how participants cope with their health challenges. The information collected provided a deeper understanding of the lived experiences of the participants. Data saturation was considered to have been reached when participants began repeating similar experiences and no new information relevant to the study objectives was obtained. Thematic analysis, including the generation of themes, was conducted after transcription, familiarization, and coding.

This qualitative approach allowed for an in-depth exploration of their experiences, offering valuable insights for future research and interventions. Interviews were held in a private space to guarantee privacy and comfort for participants. They could choose to communicate in English or local languages like Fante or Twi. Each interview lasted between 10 and 40 minutes on average. To record the information, both audio recordings and written notes were taken, with permission from the participants. The research team underwent training to manage sensitive discussions, ensuring that any emotional discomfort during the interview was properly handled.

### Interview guide development and piloting

The interview guide was developed based on a comprehensive review of literature focusing on the lived experiences and coping strategies of individuals with chronic renal failure. The guide included open-ended questions designed to elicit detailed, personal narratives aligned with the phenomenological approach of the study. To ensure clarity and relevance, the guide was piloted with nine eligible patients (not included in the final sample), and minor revisions were made based on their feedback. These changes improved the clarity, flow, and cultural sensitivity of the questions.

**Sample questions included:**

- Can you describe your experience living with chronic renal failure?
- What thoughts went through your mind when you were first told you needed dialysis?”
- How has the disease affected your daily life, relationships, or employment?”
- What coping strategies help you manage emotionally or physically?

### Recruitment period

Recruitment for the study began on April 29th, 2024, and concluded on October 11th, 2024. The extended duration of data collection was due to time and financial constraints. Additionally, some audio recordings were corrupted and incomplete, requiring the research team to revisit certain participants for data collection.

### Ethics statement

Approval for this study was granted by the University of Health and Allied Sciences Review Ethics Committee (UHAS-REC B.15 [113]23–24) and the research boards of Ho Teaching Hospital. Consent was obtained from relevant departments and censer heads at the hospitals prior to data collection. Detailed information regarding the study’s goals, methods, potential risks, and benefits was explained to participants in a manner they could understand. They were informed of their right to leave the study at any point without repercussions. After receiving the information, participants were asked to indicate their agreement through a signature or thumbprint on consent forms, with a witness present for those who were unable to read or write. To protect participants’ identities, pseudonyms were used in place of real names. All data was safeguarded by encrypting audio recordings and storing them in a secure system called ‘My Lockbox,’ while typed notes were kept in the same encrypted storage.

Physical copies were locked away in cabinets that only the research team could access. Given the emotional sensitivity of the study, special care was taken to support participants. The research team collaborated with the facility’s mental health officers, and one mental health officer from the research team was present to address any emotional distress that might arise. Participants were informed that they might experience emotional discomfort during the interviews and reassured that a counsellor would be available to assist if needed. They were also informed that they could halt or pause the interview at any time without negative consequences. The interviews were structured to conclude with neutral topics to minimize distress. Participants were provided with information for additional support after the interview, with follow-up contact offered to ensure their well-being.

### Challenges encountered

Several challenges arose during the study, which affected the data collection process. One major issue was participants’ health conditions, which sometimes prevented them from fully engaging in the interviews. In some cases, participants experienced physical discomfort or medical complications that made it difficult to continue. Another challenge was the time constraints faced by participants due to their medical appointments. Many patients had busy schedules, which made it hard to coordinate interview times. Additionally, there was an instance when a participant had to leave an interview unexpectedly due to unforeseen circumstances.

To overcome these obstacles, the research team adopted a flexible approach to scheduling, allowing for adjustments when needed. The team was also sensitive to the participants’ health needs and made accommodations to ensure they were comfortable. These efforts helped maintain the flow of data collection while minimizing disruption for the participants. Overall, the research team remained adaptable, working to ensure that the study continued smoothly despite these challenges.

### Trustworthiness

The reliability of this qualitative study was ensured by adhering to the principles outlined by Korstjens and Moser, focusing on credibility, transferability, dependability, and confirmability. Credibility was addressed through the establishment of trust with participants and the use of feedback to verify data accuracy. Transferability was supported by providing a thorough description of the study’s context, allowing the findings to be relevant to similar environments. Dependability was maintained by systematically recording the research process, ensuring consistency throughout. Confirmability was ensured by keeping detailed notes on the data and analytical procedures, minimizing bias. These elements were integrated into the study to maintain methodological rigor. By focusing on these principles, the study aimed to provide findings that are both credible and applicable. Ultimately, this approach ensured the authenticity and reliability of the results.

### Credibility

Credibility in qualitative research refers to the accuracy and trustworthiness of the findings. To ensure the credibility of this study, we employed several triangulation methods to validate the data and strengthen the reliability of our conclusions. One of the key methods used was data triangulation, which involved collecting information from various sources, including patients. This approach allowed us to compare insights from these diverse groups, which helped to confirm recurring themes and key issues related to lived experiences, coping mechanisms, and suicidal behaviours. By cross-checking the perspectives of both patients and healthcare providers, we were able to validate the emerging themes and patterns, ensuring that the findings were not based on a single viewpoint. This increased the overall credibility of the research and provided a more comprehensive understanding of the subject. Another important method for ensuring credibility was investigator triangulation. In this study, seven independent researchers were involved in the coding and theme development process. Each researcher independently generated their own initial codes and themes, which allowed for a variety of interpretations to be explored. Once this was completed, the researchers came together to discuss and refine the codes and themes. This collaborative process helped minimize individual biases and incorporated multiple perspectives, enriching the depth of the analysis. The diversity of viewpoints in the coding and theme development process further enhanced the credibility of the findings by ensuring that no single interpretation dominated the analysis.

Furthermore, member checking was employed to ensure the accuracy of the data. Although logistical constraints prevented us from returning transcripts to participants for direct verification, we implemented an alternative approach to confirm the validity of the interview data. The research team cross-checked all transcriptions against audio recordings and handwritten notes taken during the interviews. This step was crucial for ensuring that the transcriptions accurately reflected what was said during the interviews, and it allowed us to verify the data before proceeding with analysis. Cross-referencing multiple sources of data, we ensured that the findings were grounded in accurate information.

Additionally, the research team maintained a transparent and systematic approach to data handling, with clear documentation of each step in the process. This allowed for easy review and verification of the methods used, providing further assurance of the rigor and reliability of the research. Regular team meetings were held to discuss and refine the data analysis, ensuring alignment among all members and preventing any biases from influencing the interpretations. These methods combined to create a robust and trustworthy dataset, ultimately ensuring the credibility of the findings. Through the use of triangulation techniques and careful attention to detail, we were able to produce reliable and accurate results that reflect the experiences and perspectives of the participants.

### Transferability

Transferability refers to the degree to which the findings of a study can be applied to other contexts or populations. In this study, we ensured transferability by providing a detailed description of the study’s context and participant demographics, allowing readers to assess whether the findings could be relevant in other settings. We included comprehensive contextual information about the selected teaching Hospital, including its history, operations, and geographical location. This information enables readers to evaluate whether the findings could be applied to other hospitals or healthcare environments with similar characteristics. Additionally, we outlined the socio-demographic characteristics of the participants, such as their age, gender, education, and occupation. This information gives readers a clearer understanding of the diversity of the sample, allowing them to determine whether the findings might apply to other patient populations in different settings.

We also included thematic network figures that summarize the key findings of the study, offering a clear overview of the main themes identified. These network figures help contextualize the results and make it easier for readers to compare the findings with those from other studies. Additionally, the interview guides used during data collection are described, outlining the structure of the interviews and the types of questions asked. Through these efforts, we enhanced the study’s transferability and provided the necessary information for others to assess the relevance of the findings in different healthcare environments.

### Dependability

Dependability refers to the consistency and reliability of the study’s findings. To ensure dependability, we implemented several strategies to maintain the integrity of the research process. One key approach was the documentation of the research process, where every step of the study, from design through data collection to analysis, was carefully recorded. This level of documentation ensured that the process was transparent and accountable, allowing other researchers to understand the rationale behind each decision and replicate the study if needed. We also maintained an audit trail throughout the study. This involved keeping a detailed record of all decisions made during the research, including the inclusion of specific themes, adjustments to interview guides, and any modifications to data analysis procedures.

By keeping track of these decisions, we were able to trace how the findings emerged from the data and ensure that the research process was systematic and reproducible. This also provided a clear, documented path for validating the findings. Furthermore, we developed a codebook to guide the coding process. This codebook outlined the initial codes, their definitions, and the rules for applying them, which helped ensure consistency across the team.

The codebook was a crucial tool for maintaining reliability in how data was analysed and coded, helping to minimize discrepancies and ensuring that the analysis remained aligned with the study’s objectives. Together, these strategies careful documentation, an audit trail, and the use of a codebook helped to establish the dependability of the study’s findings. They ensured that the research process was transparent, systematic, and reliable, providing a strong foundation for the conclusions drawn from the data.

### Confirmability

Confirmability refers to the extent to which the findings of the study accurately reflect the participants’ perspectives, without being shaped by the researchers’ biases or preconceptions. To ensure confirmability in our study, we implemented several strategies. One of the key approaches was reflexivity, which was integral to our data analysis process. The researchers remained mindful of their own biases, backgrounds, and assumptions, reflecting regularly on how these factors might influence the research and interpretation of the findings. This allowed us to maintain awareness of potential biases and ensure that the conclusions were based on the data itself, rather than shaped by personal perspectives. Any biases identified were documented and monitored throughout data collection and analysis, to preserve the objectivity of the findings.

Independent coding was another important strategy used to minimize researcher bias. Four researchers by EAB, PYA, AK, and JK each independently coded the data, bringing diverse perspectives to the analysis. When disagreements arose during the coding process, they were resolved through discussion and consensus approach during weekly team meetings, ensuring that the final codes and themes were the result of collaborative decision-making. In instances where consensus could not be achieved, a third senior qualitative researcher was consulted to mediate and finalize the coding decision. This collaborative approach ensured reliability and rigour in theme development.

Additionally, we employed an audit trail and maintained transparency throughout the research process, as outlined under dependability. The audit trail documented each step and decision made during the study, providing a clear record that could be traced back to the raw data. This approach helped to ensure that the findings were grounded in the data and not influenced by external factors or researcher bias. These strategies contributed to the overall confirmability of the research, allowing us to confidently assert that the findings reflect the participants’ true experiences and perspectives.

### Reflexivity and potential bias

We acknowledge potential biases that may have influenced the findings of this study. First, participant recruitment was limited to patients attending a specific teaching hospital, potentially excluding individuals with differing experiences, especially those in private facilities or rural settings. This may limit the representativeness of the sample. Second, the interviewers’ professional background in health and social science research may have shaped how participants responded, particularly if they perceived the interviewer as aligned with the healthcare system. To mitigate this, interviews were conducted with empathy, neutrality, and culturally appropriate language. Third, in terms of analysis reflexivity, researchers engaged in regular debriefings and reflexive journaling to reflect on how their own assumptions, values, and positionalities might shape interpretation of the data. Independent coding by multiple team members, the use of an audit trail, and consensus-building meetings further reduced the influence of individual bias. These strategies were essential in enhancing transparency, reducing subjectivity, and improving the trustworthiness of the findings.

### Data analysis

The data were analysed using reflexive thematic analysis, guided by Braun and Clarke’s six-phase framework. All interviews were transcribed verbatim and carefully proofread to ensure accuracy. Non-English interviews were translated into English, and transcripts were cross-checked with field notes and audio recordings for data integrity. Due to logistical constraints, member-checking with participants was not feasible.

Familiarisation with the data involved repeated reading of transcripts. Preliminary coding was conducted by EAB, followed by independent confirmatory coding by PYA, AK, and JK to enhance analytical rigour. A comprehensive codebook was constructed based on consensus and used to guide subsequent coding. Discrepancies in coding were discussed and resolved through collaborative meetings until agreement was reached. Codes were then grouped into sub-themes and overarching themes based on conceptual coherence.

While this process was systematic, it also involved interpretive reflexivity. The research team reflected on their own backgrounds academic training in public health and clinical work within Ghanaian health systems and considered how these perspectives may have shaped interpretation. Regular team debriefings encouraged critical examination of assumptions and helped surface latent meanings in participants’ narratives.

Themes were interpreted in relation to both the socio-cultural context of chronic illness in Ghana and existing literature on patient coping, stigma, and resilience. Issues of power dynamics in patient–provider interactions and the social meanings of dialysis were considered in the interpretation of themes. The final themes were reviewed and refined by the entire team. Thematic coding was supported by ATLAS.ti version 7.5.7. Quotations from participants were used to illustrate themes, and frequency tables summarised participants’ socio-demographic characteristics.

## Result

### Socio-demographic characteristics of patients

Table 1 presents the socio-demographic characteristics of the participants. Out of the total participants (120), 41 (34.2%), were between aged 31–50 years, followed by 40 (33.3%) aged 25–30 years, and 39 (32.5%) aged 51 years and older. Among the total respondents, 70 (58.3%) were male, while 50 (41.7%) were female. In terms of ethnicity, the Akan group was the most represented, with 48 participants (40%), followed by Ewe with 39 (32.5%) and Ga-Dangme with 36 (30%). With respect to educational level, 40 (33.3%) had completed Senior High School (SHS), Senior Secondary School (SSS), or the General Certificate of Education (GCE) Ordinary Level (O’ Level), 41 (34.2%) had Junior High School (JHS)/primary education, and 39 (32.5%) had tertiary education.

**Table 1:** Socio-demographic characteristics of patients.

Variables	Frequency (N = 120)	Percent (%)
**Age**		
25–30	40	33.3
31–50	41	34.2
51 years and older	39	32.5
**Sex**		
Male	70	58.3
Female	50	41.7
**Ethnic Group**		
Akan	48	40.0
Ga-Dangme	36	30.0
Ewe	39	32.5
**Level of education**		
SHS/SSS/O’level	40	33.3
JHS/Primary School	41	34.2
Tertiary	39	32.5
**Religion**		
Christian	70	58.3
Muslim	50	41.7
**Marital status**		
Never Married	50	41.7
Married	30	25.0
Separated	20	16.7
Widowed	20	16.7
**Employment Status**		
Employed	80	66.7
Unemployed	40	33.3
**Ownership of Health insurance**		
Yes	120	100
**Duration of Diagnosis**		
>6 months	30	25.0
12 months to 24 months	80	66.7
More than 24 months	10	8.0

*SHS = Senior High School; SSS = Senior Secondary School; O’ Level = Ordinary Level.

With respect to religion, 70 (58.3%) identified as Christians, while 50 (41.7%) identified as Muslims. Marital status revealed that 50 (41.7%) had never been married, 30 (25%) were married, 20 (16.7%) were separated, and 20 (16.7%) were widowed. Employment status showed that 80 (66.7%) were employed, while 40 (33.3%) were unemployed. All 120 participants (100%) reported having health insurance coverage. Regarding the duration of diagnosis, 30 (25%) had been living with the condition for more than six months, 80 (66.7%) for 12–24 months, and 10 (8%) for more than 24 months.

### Thematic analysis on lived experiences of chronic renal failure

The findings of this study presented using thematic networking in ATLAS.Ti, explore the lived experiences of patients with chronic renal failure at three teaching hospitals in Ghana. The main theme identified is Lived Experience, with sub-themes and codes providing detailed insights. The sub-theme: Instantaneous Response includes codes such as Unpleasant, Terrible, Heartbreaking, Agonizing, Sorrowful, Astonishment, and Bewilderment. The sub-theme: Shifting Emotional States reflects Suicidal Ideation and Persistent Depression and Sadness. The sub-theme: Chronic Emotional Challenge includes codes like Helplessness and Depression, Anxiety and Stress, and Suicide.

The sub-theme: Physical Challenge is represented by the codes Financial Burden and Difficulty Accessing Medication. These sub-themes and codes collectively offer a comprehensive understanding of the emotional and practical difficulties faced by patients with chronic renal failure.

While Shifting Emotional States reflected the transient, fluctuating emotions participants experienced shortly after diagnosis such as denial, fear, and confusion Chronic Emotional Challenge referred to more persistent and entrenched mental health difficulties, including ongoing depression, emotional exhaustion, and hopelessness that continued even after patients had begun adapting to treatment routines.

### Lived experiences of chronic renal failure

The findings of this study provide insight into the lived experiences of patients with chronic renal failure at three teaching hospitals in Ghana. The instantaneous response to the diagnosis is often described as unpleasant, terrible, heartbreaking, agonizing, and sorrowful, with patients expressing feelings of astonishment and bewilderment. Over time, these initial emotional reactions give way to shifting emotional states, including suicidal ideation and persistent depression and sadness. The emotional toll of chronic renal failure leads to chronic emotional challenges, where patients commonly report feelings of helplessness and depression, anxiety and stress, and, in some cases, thoughts of suicide.

In addition to the emotional struggles, patients also face significant physical challenges, particularly in the form of a financial burden and difficulty accessing medication, which further complicates their condition and contributes to their overall distress. For instance, a patient had this to say: *When I was first diagnosed, it felt unpleasant, like something I couldn’t even comprehend. The news was terrible, and I felt like my whole world was turned upside down. It was heartbreaking to think about the impact on my family. I was left in complete astonishment and bewilderment, unsure of how to cope with the sudden change* (Patient, Female).

*When the doctor told me I had renal failure, it was like a blow to my heart—unpleasant and shocking. The heartbreaking reality of it hit me hard, and I felt sorrowful for what I was going through. I was in astonishment, not fully understanding how my life had changed so quickly. I was left in complete bewilderment, with no idea of how to face the future.* (Patient, Male)

Another patient also stated

*Hmm…. Erh, you see, it was not easy oo my sister and even now is more difficult for me. Honestly the moment I was told I had chronic renal failure, I felt agonizing pain, both physically and emotionally. It was sorrowful to accept that my life would never be the same. I was in astonishment, thinking that this couldn’t be happening to me. It was a terrible feeling, and I was left feeling completely bewildered about what to do next.* (Patient, Male)

### Shifting emotional states

The Shifting Emotional States captures the early to intermediate emotional transitions participants experienced shortly after diagnosis such as denial, confusion, hope, and fear which were often transient and fluctuating. These were largely driven by adjustments to the new diagnosis and uncertainty about the future. The study explores that, as patients with chronic renal failure face their diagnosis, they experience significantly shifting emotional states. Many individuals reported feeling persistent depression and sadness, which heavily influenced their daily lives and overall mental health. Some patients also expressed thoughts of suicidal ideation, feeling overwhelmed by the emotional toll of their condition. For instance, a patient said:

*I wake up every day feeling like I’m stuck in a dark place. The sadness doesn’t leave me, and sometimes, I wonder if it’s even worth continuing. The emotional pain is unbearable, and I feel like I can’t take it anymore. It’s hard to find hope when everything feels so hopeless and sometimes, I want to commit suicide*. (Patient, Male)

Similar expressions of hopelessness and suicidal thoughts were echoed by other participants

*I feel like I’m drowning in sadness every day, and it never seems to end. The depression takes over, and I can’t escape it. Sometimes, I even think that ending it all might be better than living with this constant pain and hopelessness. The thought of suicide crosses my mind because I just can’t see a way out of this dark place.* (Patient, Female)

Other participants focused on the chronic exhaustion and the weariness of carrying this emotional burden into an indefinite future. Beyond these experiences of despair, some patients described emotional detachment and a loss of connection to their previous selves:

*“I don’t feel like the same person anymore. Since the diagnosis, it’s like I’m just going through the motions eating, sleeping, existing. I used to be active and hopeful, always planning for the future, but now that energy is gone. I feel emotionally numb, like a part of me has shut down completely, and I’m just watching life happen around me.” (Patient, Male)*

These accounts highlight not only the presence of suicidal ideation, but also the varied emotional nuances from acute psychological pain and emotional paralysis to numbness and identity disruption that patients with chronic renal failure often endure.

### Chronic emotional challenges

**Fig 1:**
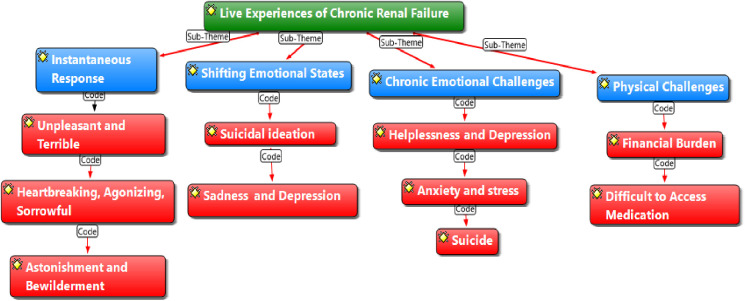
Thematic analysis network on lived experiences of chronic renal failure.

In contrast, “Chronic Emotional Challenges” represents more entrenched, persistent emotional states, such as long-term depression, anxiety, and emotional fatigue, which developed as participants grappled with the prolonged burden of living with chronic renal failure. These were described as sustained and debilitating, rather than shifting or reactive. Living with chronic renal failure poses significant emotional challenges for patients, often extending beyond the initial diagnosis to affect their long-term mental well-being. These challenges include persistent feelings of depression, anxiety, and stress, which can become overwhelming as patients navigate the demands of their condition which can be reflected in Fig 1 under the sub theme of chronic emotional challenges. Many patients describe a sense of helplessness and emotional exhaustion that worsens over time, impacting their ability to cope with daily life. Some patients shared their experiences of these chronic emotional challenges. One patient said: “*There are days when the depression feels unbearable. The sadness weighs on me constantly, and I feel like I can never escape it. I wake up feeling anxious, and the stress of managing this condition never goes away.”* (Patient, Male). Another patient *expressed: “I feel helpless most of the time, like I have no control over my life anymore. The anxiety and stress make it hard to focus on anything else, and sometimes I wonder if I can keep doing this. It feels like the struggle is never-ending, and it’s hard to find hope.”* (Patient, Female).

### Physical challenges

The study also explores the physical challenges faced by patients with chronic renal failure, particularly the financial burden of treatment and the difficulty accessing essential medications. For many patients, the high cost of dialysis sessions adds immense pressure to their already strained resources, forcing difficult choices between basic needs and medical care. Patients also reported challenges in finding and affording medications critical to their treatment, further complicating their condition. One patient shared: *“Getting the medication I need is a constant struggle. It’s either too expensive, or I have to travel long distances to find it.”*(Female, Patient) This statement reflects the immense strain caused by the cost and availability of essential treatments. The financial pressure is compounded by the recurring expense of dialysis, which many patients find unaffordable. Another patient explained: *“The financial strain from both the cost of dialysis and medication is overwhelming, and it feels like there’s no end to the challenges.” (Patient, Female)*

*Obtaining the medication I need is an ongoing challenge; it’s often too expensive, and when it is available, I have to travel long distances to both the pharmacy and the hospital, which only adds to my expenses. The cost of dialysis makes it even harder because I need it regularly to stay alive, and it’s not something I can skip. Sometimes, I have to decide whether to pay for my treatment or buy food for my family, and that decision is heartbreaking. I feel like I’m constantly borrowing money or begging for help just to survive. The financial burden is overwhelming, and it feels like no matter how hard I try, I can never catch up. Living like this every day is exhausting, and I don’t see how things will get better.“* (Patient, Male)

### Coping strategies adopted by patients

**Fig 2:**
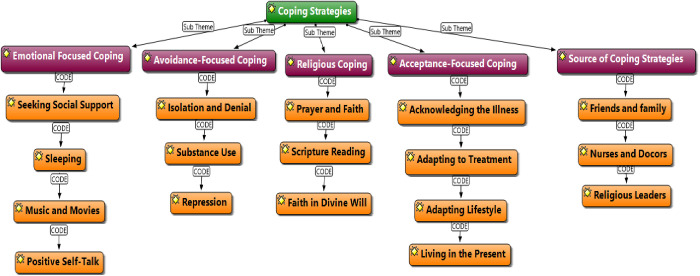
Thematic network analysis on coping strategies adopted by patients.

Fig 2 presents the coping strategies employed by patients, as represented through thematic networking. The study explores how patients with chronic renal failure manage the challenges of their condition, highlighting the diverse approaches they use to navigate the emotional, physical, and spiritual burdens of their illness. The findings reveal that patients rely on four main strategies: emotional coping, avoidance-focused coping, religious coping, and acceptance coping. These strategies, captured through the thematic network, showcase how individuals adapt to their circumstances and seek relief amidst the difficulties they face.

### Emotional coping strategies

From the coping strategies, emotional coping was explored as a keyway for patients with chronic renal failure to manage the emotional challenges associated with their condition. These strategies include positive self-talk, where patients encourage themselves to stay hopeful despite the overwhelming circumstances, and seeking social support from family, friends, or peers to reduce feelings of isolation. Many patients also engage in music and movies as a temporary escape from the stress, while others find solace in sleeping, using rest as a way to recharge both emotionally and physically. One patient shared: “*I try to stay positive and remind myself that this is just a phase. Talking to my family helps a lot; they keep me going and make me feel like I’m not alone in this”* (Patient, Male). Another patient also stated: *“Whenever I feel overwhelmed, I listen to music or watch a movie to take my mind off things. It helps me forget, even if it’s just for a little while.*” (Patient, Male).

*“I try to stay optimistic and remind myself that this is just a temporary phase. I keep telling myself I can get through this, even when it feels overwhelming. Speaking to my family really helps; they offer me support and remind me that I’m not facing this alone. Sometimes, I write down my thoughts to clear my head and unload my emotions. It’s a way to express how I’m feeling without burdening others.”* (Patient, Female)

Another patient explained:

*“When I feel weighed down by everything, I turn to music or watch movies to get my mind off things. It allows me to escape, even if only for a short time, and brings me a sense of relief. These activities help me feel like I’m still in control, not just defined by my illness. Music, in particular, soothes me and takes me back to better moments in life before my illness took over. It’s my way of finding peace in the midst of the chaos.”* (Patient, Female)

This narrative reveals how engaging in familiar activities like listening to music or watching movies offers emotional comfort and a temporary reprieve from the overwhelming realities of chronic illness.

### Avoidance-focused coping

The study highlights avoidance-focused coping as a strategy adopted by some patients with chronic renal failure to distance themselves from the reality of their condition. Many patients reported withdrawing from social interactions, opting for isolation as a way to avoid confronting their illness. For instance, patient shared*: “erh… you see er…. I stopped seeing my friends because I didn’t want to answer questions about my health. It’s easier to stay away and deal with it on my own.”* (Patient, Female). One patient stated: *“I’ve distanced myself from everyone, even my closest friends. It’s not that I don’t care about them, but I feel like they can’t understand what I’m going through, and I don’t want to burden them”.*(Patient, Female) Another patient shared a similar experience, emphasizing the deep emotional pain tied to their sense of isolation*:*

*“I prefer to stay alone because being around people reminds me of the life I used to have before my diagnosis.” They explained how seeing others live normal, healthy lives serves as a constant reminder of what they have lost. “It’s painful to watch others smile, work, and carry on with ease while I’m struggling every day to manage my illness,” they added.* (Patient. Male)

The patient further mentioned that withdrawing from others feels like a form of self-protection, shielding them from pity or uncomfortable questions about their condition. However, they admitted that this isolation also leaves them feeling lonely, deepening their emotional burden and sense of separation from the world they once knew. Denial also emerged as a common coping mechanism, with patients refusing to fully accept the severity of their condition. A participant said*: “I told myself for months that the doctors were wrong. I couldn’t accept that this was happening to me.”*(Patient, Male). Another patient also shared: “*I refused to believe it when the doctors told me. I kept thinking there must have been a mistake, that they mixed up my test results.”* (Patient, Male). They explained that for months, they avoided follow-up appointments and ignored symptoms, hoping the condition would disappear on its own. *“I didn’t want to accept that this was happening to me. It felt easier to pretend everything was fine rather than face the truth.” (Patient, Female).* However, the patient admitted that this denial eventually delayed treatment, worsening their physical and emotional challenges. Despite the temporary comfort it provided, the avoidance left them feeling unprepared to deal with the realities of their illness. *For instance, a patient said:*

*“I kept telling myself it wasn’t real, that the doctors must have gotten it wrong. I ignored their advice and avoided going back to the hospital because I didn’t want to hear more bad news. By the time I finally accepted it, my condition had gotten worse, and I realized how much time I had wasted.”* (Patient, Male)

For some, substance use became a way to manage the overwhelming emotional strain of chronic renal failure. One patient confessed: “*When it all feels like too much, I turn to alcohol. It helps me forget, even if it’s just for a little while*.” (Patient, Male). They explained that consuming alcohol offered a temporary escape from the relentless worries about their health and the burdens of treatment. “*In those moments, it’s like I can pretend everything is normal again,”* (Patient, Male), *they* added. However, the patient acknowledged that this approach often left them feeling worse afterward, both physically and emotionally, as the problems they tried to avoid remained unresolved. For example, another patient shared: *“I thought drinking would help me cope, but it only made me feel more isolated. Afterward, I’d feel guilty and sick, and the reality of my situation would hit even harder.” (*Patient, Male). This narrative illustrates the destructive cycle of substance use as a coping mechanism, providing fleeting relief but exacerbating the long-term challenges of living with chronic illness.

Repression was another common coping strategy adopted by patients with chronic renal failure. One participant shared: *“I don’t talk about how I feel, not even with my family. I keep it all inside because I don’t want to seem weak* (Patient, Female*).”* They explained that expressing vulnerability felt uncomfortable, as they feared burdening loved ones or being perceived as incapable of managing their condition. *“I’ve learned to hide my pain and put on a strong face, even when I’m breaking inside,”*(Patient, Female), they added. While repression allowed them to maintain an outward appearance of strength, it often led to a buildup of emotional stress and feelings of isolation. Another patient echoed this sentiment, stating: *“Holding it in feels like the only option, but some days, the weight of it all becomes unbearable.”.” (*Patient, Male). These accounts reveal how repression may serve as a short-term defence mechanism but can intensify the psychological challenges faced by patients.

### Religious coping strategies

The study also explored how patients with chronic renal failure utilized religious coping strategies to manage their illness. Many patients drew strength from their faith, engaging in spiritual practices to help them cope with the emotional, physical, and psychological challenges they faced. A patient shared: *“Being part of my church has given me a sense of belonging. They pray for me and encourage me when I feel weak.”* (Patient, Female). Prayer and faith emerged as a central practice, with patients turning to prayer to seek comfort and guidance. *“I pray every day, asking God for strength to face another day. It gives me peace when everything feels overwhelming,”* said another Female Patient.

*“Whenever the pain gets too much, I turn to prayer. I just sit quietly and pour my heart out to God, asking for strength to keep going. In those moments, it’s just me and Him, and I let everything out. Sometimes I cry while praying, but it feels like a weight is lifted off my shoulders. Prayer is the only thing that gives me the strength to face another day*.” (Patient, Male)

Others relied on scripture reading to find meaning and hope in their circumstances. A patient explained: “*Reading the Bible reminds me that God has a plan for me, even if I don’t understand it right now.*” (Patient, Female). They described how specific verses brought comfort and reassurance during moments of doubt and fear. Another patient shared: *“Whenever I feel overwhelmed, I turn to the Psalms. It feels like they were written just for me.”* (Patient, Female). For these patients, scripture reading provided a sense of purpose and faith, helping them to endure the challenges of their illness.

*“When I read certain passages, I feel like God is speaking directly to me, reassuring me that I’m not alone.” “It’s like a reminder that I have a purpose, and no matter how tough it gets, God’s plan is bigger than this illness.” “The words were meant for me, giving me strength to keep going.” “When I read those words, it reminds me that there’s more to my life than just this illness.” “It gives me the courage to face another day.”* (Patient, Male)

Faith in divine will also provide reassurance to many patients. One participant reflected: *“I believe that whatever happens is God’s will”. Trusting in Him gives me the courage to keep going.”* (Patient, Male). These religious practices not only offered emotional relief but also reinforced patients’ resilience in coping with the complexities of their illness. For instance, a patient said

*“ Ok… for me… erh…. err…. I will say I believe that whatever happens is God’s will though in those moments of doubt, I remind myself that God knows what’s best for me.” “Even when it feels like things are falling apart, I have faith that God is guiding me.” “It’s this belief that helps me get through the toughest days.” “No matter the challenges, I trust that God has a plan for me.”* (Patient, Male)

### Acceptance coping strategies

The study also explored the coping strategies patients with chronic renal failure employed in accepting their condition. One key strategy was acceptance coping, where individuals acknowledged their illness and adapted to the challenges it brought. This approach involved adapting to treatments, modifying lifestyle choices, and focusing on living in the present moment. The findings highlight how patients’ ability to accept their illness, despite its difficulties, enabled them to better cope with their physical and emotional struggles. Through this acceptance, patients found resilience and a sense of peace in managing their condition. For instance, some patients stated:

*“I’ve come to accept that this is my reality now, and I try to live with it.” One patient expressed how acknowledging the illness allowed them to focus on living their life as best as they could. “At first, it was hard to accept, but now I know I have to adapt if I want to survive.” The patient shared how adapting to the treatment schedule and changing their lifestyle became a necessary part of their routine. “It’s not easy, but I’ve learned to live in the present, one day at a time.”* (Patient, Male).

For this patient, acceptance wasn’t giving up; rather, it was finding peace in the current circumstances. Another patient said *“I can’t change my situation, so I’ve learned to accept it. There’s no point in fighting it.”* (Patient, Female). This patient explained how acceptance helped them manage their emotions and the daily struggles that came with treatment. The patient continues to state that, *“I’ve had to adapt to the dialysis and the diet restrictions, but I’ve made peace with it”.* (Patient, Female)*.* They noted that acceptance allowed them to stop feeling defeated by the condition. A female patient also said, *“Living in the moment helps me focus on what I can control, and that’s all I can do.”*

*“At first, I resisted the idea that I had to live with this, but now I’ve accepted it.” A patient revealed how adapting to the illness and its treatments became part of their coping process. “I had to change my diet, my routine, and my mindset, but it’s helping.” They acknowledged that the changes were not easy, but they allowed themselves to focus on the positives. “Now, I try to appreciate each day I have, even with all the challenges.”* (Patient, Male).

### Sources of coping strategies

The study revealed that patients with chronic renal failure relied on various sources of coping to manage their challenges. Friends and family provided emotional and practical support, offering comfort during difficult times. Religious leaders played a crucial role, offering spiritual guidance and reassurance, which strengthened patients’ faith and resilience. Nurses and doctors were also vital sources of coping, as their care and encouragement helped patients navigate the complexities of their treatments. These support systems collectively contributed to the patients’ ability to endure and adapt to their condition. For example, A female patient said

*“My family has been my rock through all of this. They are always there to support me, whether it’s helping with my medication or just listening when I need to talk. Without them, I don’t know how I would have made it through some of the toughest moments”.* (Patient, Male)*.*

Another patient shared,

*“My pastor always reminds me that God has a plan for everyone, including me. He tells me to hold on to my faith, even when it feels like there’s no hope. His words give me the strength to keep going, even on the hardest days.”* (Patient, Female)

*“The nurses are like family to me now. They not only care for me but also encourage me to stay strong and keep fighting. Their kindness and support make such a difference in my journey. Sometimes, it’s the small things they do, like a smile or a kind word, that lift my spirits. They understand my struggles in ways that others might not and always make me feel like I’m not alone. Knowing that they genuinely care gives me hope and the strength to face another day.”* (Patient, Male)

## Discussion

This phenomenological study explored the lived experiences and coping strategies of patients with chronic renal failure (CRF) across three teaching hospitals in Ghana. The study illuminated the multidimensional burdens patients face emotional, physical, financial, and social while also revealing adaptive and maladaptive coping mechanisms. Drawing on Lazarus and Folkman’s Stress and Coping Theory and the Interactive Stress Theory (IST) [21,22], the analysis extends beyond individual psychology to incorporate environmental, systemic, and relational factors influencing patients’ adjustment to illness. The study reveals the deeply challenging and multifaceted journey of patients with chronic renal failure, characterized by emotional and physical hardships. Initial reactions to the diagnosis were overwhelmingly negative, with patients describing feelings of disbelief, sorrow, helplessness, and shock. These reactions, often accompanied by a sense of bewilderment, highlight the difficulty patients face in processing the diagnosis and its implications. Over time, the emotional toll evolved into more sustained and complex states, as patients reported persistent depression, sadness, and suicidal ideation.

This shift, captured under the sub-theme Shifting Emotional States, underscores the immense mental health burden imposed by chronic renal failure, with some patients expressing those suicidal thoughts offered an escape from their suffering. These shifting emotional states resonate with findings by Abu Bonsra et al. [22], where emotional dysregulation among cancer patients was similarly attributed to poor prognosis, limited social buffers, and perceived helplessness. From the IST perspective, the emotional distress experienced by patients is shaped not only by the illness but also by external factors such as financial insecurity and inconsistent care environments. The findings also align with existing literature, for instance, [23] similarly observed significant emotional strain among patients coping with the irreversible nature of disease progression. [24] also noted that individuals undergoing haemodialysis often experience life limitations and psychological uncertainty. The persistent sorrow and thoughts of suicide reported in this study emphasize the need for comprehensive mental health interventions to support patients’ emotional well-being.

In addition to emotional struggles, physical challenges such as the financial burden of treatment and difficulty accessing medications exacerbate patients’ distress, creating a cycle of hardship. The structural inequities embedded within Ghana’s healthcare financing system intensified patient distress. Many participants highlighted the high cost of haemodialysis and medication, which often forced trade-offs between basic sustenance and healthcare. Despite Ghana’s National Health Insurance Scheme (NHIS), coverage for renal therapy remains insufficient a reflection of systemic policy failure. This supports earlier critiques [22], that current health financing mechanisms disproportionately burden chronic illness patients, especially in low-resource regions. The call for subsidization policies made by study participants mirrors similar calls by Abu Bonsra et al. [22], who identified cost as a critical barrier to sustained care and adherence among cancer patients. These findings also resonate with [25], who highlighted the socio-economic difficulties affecting not only patients but also their families and social networks. Participants in this study described the cost of dialysis and medications as overwhelming, with some having to choose between basic necessities and essential medical care. The progression from initial shock to chronic emotional and physical challenges provides an in-depth understanding of the psychological trajectory of chronic renal failure patients. This progression highlights the dynamic and evolving nature of their lived experiences, reinforcing the need for holistic care approaches that address both medical and psychosocial dimensions.

Furthermore, the study reflects the broader impact of chronic renal failure on families and caregivers, emphasizing the interconnectedness of patients’ struggles with their social dynamics. [25] similarly observed the psychological and social effects of chronic kidney disease on relatives and friends. This shared burden underscores the systemic nature of challenges associated with managing chronic renal failure and the importance of supportive interventions for both patients and their families. This study’s findings imply the importance of integrating mental health services into the care of chronic renal failure patients, given the profound emotional challenges such as depression, anxiety, and suicidal ideation they face. Addressing these psychological burdens through counselling and psychosocial support is essential to improve their quality of life. The significant financial strain associated with dialysis and medication underscores the need for policies to subsidize treatment costs and enhance access to essential healthcare resources. Moreover, the interconnected impact on families calls for family-centred interventions to support caregivers and strengthen the social support system surrounding patients. Educational initiatives tailored to patients and families can further empower them to manage the condition more effectively, reducing distress and improving long-term outcomes.

This study explored the coping strategies employed by patients with chronic renal failure, revealing diverse approaches to managing their condition. The strategies identified emotional coping, avoidance-focused coping, religious coping, and acceptance highlight the complex ways patients adapt to the physical, emotional, and spiritual burdens of their illness. Emotional coping strategies, such as positive self-talk, seeking social support, and engaging in leisure activities, helped foster hope and reduce feelings of isolation, allowing individuals to maintain emotional resilience. In line with IST, patients’ coping responses were shaped by interactions between internal capacities (resilience, belief systems) and external conditions (family support, healthcare quality). Emotional and spiritual support from family, friends, and religious leaders were key protective factors. Yet, these informal safety nets are often fragile and can be unsustainable without institutional reinforcement. Social connections, particularly with family and friends, emerged as a critical source of strength, enabling patients to navigate psychological challenges.

Avoidance-focused coping, such as denial and social withdrawal, was also prevalent. While these strategies provided temporary relief, they often contributed to isolation and delayed treatment. Some patients withdrew from social interactions to avoid discussing their condition, while others turned to substance use as an escape, which worsened their challenges. Religious coping, including prayer, scripture reading, and faith, played a significant role in helping patients manage their illness by providing comfort and reinforcing resilience. Acceptance emerged as another vital coping strategy, with patients who acknowledged their condition finding it easier to manage both the physical and emotional demands of their illness. The study’s findings align with existing literature on coping strategies for chronic renal failure, particularly in Ghana. Patients with chronic renal failure use a range of strategies, including emotional, task-oriented, and avoidance-oriented approaches, as described by [26]. Like [27], the study emphasizes the importance of seeking support, managing emotions, and practicing self-care. Religious coping, a central theme in the study, reflects findings from [28], who noted the role of faith in managing illness in Ghana.

Many patients rely on family and friends for support, which aligns with the crucial role of caregivers described by [29], as strong social networks reduce isolation and enhance emotional resilience. Negative coping behaviours, such as avoidance and distancing, echo findings from [30], with these strategies potentially contributing to emotional isolation and hindering illness management [12]. The effectiveness of coping strategies varies based on treatment modality, time since diagnosis, and comorbidities, supporting [22,31]. Some participants described relying primarily on spiritual or passive acceptance, while others employed a broader mix of emotional, behavioural, and social strategies to manage their condition. These variations reflect the diverse ways people experience and adapt to chronic renal failure, reinforcing the importance of tailoring interventions to individual needs and contexts. This observation aligns with earlier studies [22,30,12] and underscores the value of incorporating patients lived experiences into care delivery, as recommended by [22,32,33]. Healthcare professionals should offer post-diagnosis counselling, strengthen collaboration with families, and tailor interventions to address the specific coping strategies and challenges of patients, which could improve their overall well-being and outcomes [22].

A particularly noteworthy finding in this study was the prominent use of acceptance coping, where participants acknowledged their chronic condition and consciously adapted their behaviours and mindset to live as well as possible. This aligns with findings from other research such as a study of maintenance haemodialysis patients which identified illness acceptance as a key predictor of better psychological well-being, including reduced anxiety and depression [34]. Another investigation demonstrated that applying acceptance-based strategies like Acceptance and Commitment Therapy (ACT) in end-stage renal disease patients improved adherence and quality of life [35]. These parallels confirm that acceptance is not only a meaningful cognitive-emotional response in chronic kidney disease but may also be a critical point of intervention in patient care. While many existing studies emphasize problem- or emotion-focused coping, our qualitative data suggest that a deliberate reorientation toward acceptance fosters resilience and peace. Given its documented benefits, integrating acceptance-based approaches such as ACT, mindfulness, or other psychosocial interventions into clinical protocols could enhance support for chronic renal failure patients.

The study also found that patients with chronic renal failure rely on various sources of support to cope with the physical, emotional, and psychosocial challenges of their condition. Family and friends were identified as primary sources of emotional and practical assistance, offering crucial support in managing medication, daily activities, and providing comfort during difficult times. Religious leaders also played a significant role, offering spiritual guidance and encouragement, which helped patients maintain faith and resilience in the face of adversity. Healthcare providers, including nurses and doctors, were essential sources of professional and emotional support, with their care, empathy, and encouragement helping patients navigate treatment complexities and stay motivated. These findings align with existing literature that highlights the role of external support, emotion management, and faith as key coping mechanisms for individuals with chronic kidney disease [36]. Caregivers, particularly those supporting children with chronic kidney disease, also face significant burdens, including emotional distress, family strain, and social pressures, as reported by [2,37]. These challenges underline the interconnected nature of coping strategies for both patients and their caregivers. In addition, during the COVID-19 pandemic, individuals with chronic conditions, including kidney disease, adopted diverse coping mechanisms, with active coping, emotional support, and religious practices contributing to better overall well-being [38]. This highlights the dynamic interplay of individual and external resources in fostering resilience among patients and their caregivers, emphasizing the need for integrated support systems to enhance coping outcomes.

## Conclusion

Living with chronic renal failure significantly impacts patients’ emotional, physical, and financial well-being, with many experiencing depression, anxiety, and suicidal thoughts. Despite these challenges, patients employ coping strategies such as emotional expression, avoidance, reliance on religious faith, and acceptance. The integration of traditional and spiritual practices alongside medical care emphasized the importance of culturally sensitive support. The study emphasizes the need for the Ghana Health Service to prioritize individualized interventions, post-diagnosis counselling, and greater family involvement to enhance coping effectiveness and improve patient outcomes. Training religious leaders in basic mental health first aid to provide psychosocial support and appropriate referrals. And integrating routine mental health screening into nephrology clinics to identify and address depression, anxiety, and suicidal ideation early.

### Strengths and limitations of the study

This study explored the lived experiences and coping strategies of patients with chronic renal failure across three selected teaching hospitals in Ghana. It revealed significant emotional, physical, and financial challenges faced by patients and highlighted the coping mechanisms employed, such as religious reliance, emotional support, and acceptance, influenced by socio-cultural and family factors. One major strength of the study is its contextual relevance, as it provides rich insight into the lived realities of chronic renal failure patients in a Ghanaian healthcare setting. The use of in-depth qualitative interviews with 120 participants a substantial sample size by qualitative standards allowed for a comprehensive exploration of nuanced patient experiences.

However, some limitations must be acknowledged. First, the use of purposive sampling and the focus on patients from tertiary care facilities may limit the generalizability of findings to other populations, such as those receiving care in rural or district hospitals. Additionally, the reliance on self-reported data introduces potential recall and social desirability biases. Another noteworthy limitation is that all participants were covered by the National Health Insurance Scheme (NHIS). While this provided a consistent baseline for assessing treatment challenges, it may not reflect the heightened financial and emotional distress experienced by uninsured patients. Future research should include participants with varied insurance statuses and care settings to capture a more holistic view of the socioeconomic and psychological dimensions of coping with chronic renal failure.

## Supporting information

S1 Checklist Qualitative COREQ checklist. Completed COREQ (Consolidated Criteria for Reporting Qualitative Research) checklist outlining the methods, data collection, and analysis procedures used in the study.
(DOCX)

S1 Data Minimal qualitative dataset. (CSV)
