# Supplementary material for: Experiences and coping strategies among patients with Chronic Renal Failure (CRF) in Ghana: A phenomenological study
Source: PLOS Ment Health. 2025 Sep 19;2(9):e0000279. doi: 10.1371/journal.pmen.0000279 (PMC12798209; doi:10.1371/journal.pmen.0000279)
Supplement: S1 Checklist — Completed COREQ (Consolidated Criteria for Reporting Qualitative Research) checklist outlining the methods, data collection, and analysis procedures used in the study. (DOCX) [file pmen.0000279.s001.docx]

**Designed by Abu Bonsra Emmanuel (FNBSPH-MRCG)**

**COREQ (Consolidated criteria for Reporting Qualitative research) Checklist**

This COREQ (Consolidated Criteria for Reporting Qualitative Research) checklist has been completed to accompany the manuscript titled "Experiences and Coping Strategies Among Patients with Chronic Renal Failure in Ghana: A Phenomenological Study." The checklist ensures transparent and comprehensive reporting of the qualitative study in line with established standards. It covers key aspects of the research process, including the research team and reflexivity, study design, and data analysis and reporting.

| **Topic** | **Item No.** | **Guide Questions/Description** | **Reporting.** |
| --- | --- | --- | --- |
| **DOMAIN 1: RESEARCH TEAM AND REFLEXIVITY** | | |  |
| ***Personal characteristics*** |  |  |  |
| Interviewer/facilitator | 1 | Which author/s conducted the interview or focus group? | Page 1– Emmanuel Abu Bonsra and Princess Yesutor Atsrim conducted all interviews. |
| Credentials | 2 | What were the researcher’s credentials? E.g. PhD, MD, bachelor | Page 1 – Emmanuel Abu Bonsra (BPH), Princess Yesutor Atsrim (BPH), Alex Korankye (BPH), Joyce Komesuor (PhD) |
| Occupation | 3 | What was their occupation at the time of the study? | Page 1 – All authors were public health researchers; Joyce Komesuor was a university lecturer. |
| Gender | 4 | Was the researcher male or female? | Page 5 – Emmanuel and Alex (male), Princess and Joyce (female). |
| Experience and training | 5 | What experience or training did the researcher have? | Page 5 – The interviewers had training in qualitative research and had conducted prior interviews under supervision. |
| **RELATIONSHIP WITH PARTICIPANTS** | | |  |
| Relationship established | 6 | Was a relationship established prior to study commencement? | No prior relationship existed; rapport was built at the time of consent and data collection. |
| Participant knowledge of the interviewer | 7 | What did the participants know about the researcher? e.g. personal goals, reasons for doing the research | Participants were informed about the aim of the study and the research team's academic background. |
| Interviewer characteristics | 8 | What characteristics were reported about the inter viewer/facilitator? e.g. Bias, assumptions, reasons and interests in the research topic | Researchers disclosed academic interest in patient experiences with chronic illness but had no personal or clinical involvement. |
| **DOMAIN 2: STUDY DESIGN** | | |  |
| *Theoretical framework* |  |  |  |
| Methodological orientation and Theory | 9 | What methodological orientation was stated to underpin the study? e.g.  grounded theory, discourse analysis, ethnography, phenomenology, content analysis | phenomenological approach was used to explore lived experiences. The study did not adopt a pre-existing theoretical framework but was guided by participants’ narratives, allowing themes to emerge naturally from the data. |
| **PARTICIPANT SELECTION** | | |  |
| Sampling | 10 | How were participants selected? e.g. purposive, convenience, consecutive, snowball | Purposive sampling was used to recruit individuals diagnosed with chronic renal failure. |
| Method of approach | 11 | How were participants approached? e.g. face-to-face, telephone, mail, email | Participants were approached face-to-face at the hospital during dialysis appointments. |
| Sample size | 12 | How many participants were in the study? | A total of 120 participants were interviewed. |
| Non-participation | 13 | How many people refused to participate or dropped out? Reasons? | Initially, 140 participants were invited to participate in the study; however, 20 declined due to illness, communication barriers, or time constraints. The final sample consisted of 120 participants, at which point data saturation was achieved. |
| *Setting* |  |  |  |
| Setting of data collection | 14 | Where was the data collected? e.g. home, clinic, workplace | In a private consulting room within the renal unit of a tertiary hospital. |
| Presence of nonparticipants | 15 | Was anyone else present besides the participants and researchers? | No; interviews were conducted in private with only the participant and interviewer present. |
| Description of sample | 16 | What are the important characteristics of the sample? e.g. demographic data, date | Participants ranged in age from 26 to 65, on dialysis for at least 6 months, and varied in sex, education, and employment. |
| *Data collection* |  |  |  |
| Interview guide | 17 | Were questions, prompts, guides provided by the authors? Was it pilot tested? | The interview guide was developed based on a comprehensive review of literature focusing To ensure clarity and relevance, the guide was piloted with nine eligible patients (not included in the final sample), and minor revisions were made based on their feedback. |
| Repeat interviews | 18 | Were repeat inter views carried out? If yes, how many? | No repeat interviews were conducted. |
| Audio/visual recording | 19 | Did the research use audio or visual recording to collect the data? | Yes, interviews were audio recorded with participant consent |
| Field notes | 20 | Were field notes made during and/or after the interview or focus group? | Yes, field notes were taken during and immediately after each interview. |
| Duration | 21 | What was the duration of the inter views or focus group? | – Interviews lasted between 30 and 45 minutes |
| Data saturation | 22 | Was data saturation discussed? | Yes; saturation was achieved |
| Transcripts returned | 23 | Were transcripts returned to participants for comment and/or | No; transcripts were not returned due to logistical limitations. |
| **Topic** | **Item No.** | **GUIDE QUESTIONS/DESCRIPTION** | **Reported on Page No.** |
|  |  | correction? |  |
| **Domain 3: analysis and findings** |  |  |  |
| *Data analysis* |  |  |  |
| Number of data coders | 24 | How many data coders coded the data? | Two independent researchers coded the transcripts |
| Description of the coding tree | 25 | Did authors provide a description of the coding tree? | A coding tree was developed |
| Derivation of themes | 26 | Were themes identified in advance or derived from the data? | Themes were derived inductively from the data. |
| Software | 27 | What software, if applicable, was used to manage the data? | ATLAS.ti was used for coding and thematic analysis |
| Participant checking | 28 | Did participants provide feedback on the findings? | No participant checking of findings was conducted. |
| **REPORTING** | | |  |
| Quotations presented | 29 | Were participant quotations presented to illustrate the themes/findings?  Was each quotation identified? e.g. participant number | – Yes, direct quotes were used and identified with participant numbers |
| Data and findings consistent | 30 | Was there consistency between the data presented and the findings? | Yes, findings were directly supported by quotations and thematic patterns |
| Clarity of major themes | 31 | Were major themes clearly presented in the findings? | Major themes were highlighted and described in detail |
| Clarity of minor themes | 32 | Is there a description of diverse cases or discussion of minor themes? | Yes, deviant and contrasting cases were discussed to enhance credibility |
